# Synergistic potentials of small extracellular vesicles, biomaterials, and 3D bioprinting in periodontal regeneration: a scoping review

**DOI:** 10.20517/evcna.2025.107

**Published:** 2025-12-11

**Authors:** Chenyi Zhang, Chun Liu, Andrew Liaw, Sašo Ivanovski, Pingping Han

**Affiliations:** School of Dentistry, Centre for Orofacial Regeneration, Rehabilitation and Reconstruction (COR3), Epigenetics nanodiagnostic and therapeutic group, The University of Queensland, Brisbane 4006, Australia.

**Keywords:** Small extracellular vesicles, 3D bioprinting, periodontitis, regeneration, tissue engineering

## Abstract

Periodontitis is a chronic inflammatory disease characterized by the progressive destruction of both soft (gingiva and periodontal ligament) and hard (cementum and alveolar bone) supporting tissues. The complex periodontal microenvironment often limits the effectiveness of current clinical treatments in achieving functional tissue regeneration. Although mesenchymal and immune cell-based therapies hold promise, concerns related to cell viability and immune compatibility limit their clinical translation. As a natural secretome, small extracellular vesicles (sEVs) are cell-secreted nanoparticles that deliver bioactive molecules for cell-to-cell communication to modulate immune response and promote tissue regeneration. To assess the translational readiness of sEVs therapy, this scoping review first outlines the current clinical trials of mesenchymal stem cells (MSCs)-sEVs in periodontitis, followed by a transition to preclinical application of integrating sEVs with biomaterial scaffolds to enhance localized regenerative outcomes. We then analyzed eight preclinical studies utilizing 3D bioprinted MSCs-sEVs/human umbilical vein endothelial cells-sEVs (or immune cell-derived sEVs) constructs in bone and vasculature regeneration models, and one study related to *in vitro* periodontal regeneration. These constructs exhibited improved outcomes in osteogenesis, angiogenesis, and immunomodulation, supporting their potential for future translational applications in periodontal therapy. Given the early stage of bioprinted sEVs constructs in periodontitis, we outline critical research gaps and potential future directions to overcome current technical and biological challenges. Together, this review demonstrated the translational trajectory of sEV-based strategies for periodontal regeneration. It offers a potential roadmap for utilizing sEV-based periodontal regeneration across clinical, preclinical, and biofabrication applications, highlighting their potential as next-generation, cell-free therapeutics in regenerative periodontics.

## INTRODUCTION

Periodontitis is a chronic inflammatory disease initiated by plaque biofilm, leading to progressive periodontal tissue destruction through microbial dysbiosis and complex host immune interactions^[1,2]^. The disease process involves multiple cell types, including dental mesenchymal stem cells (MSCs), osteoblasts, osteoclasts, osteocytes, stem cells, T cells, and macrophages, where they respond to microbial pathogens and tissue damage by releasing pro-inflammatory cytokines that drive the breakdown of important periodontal tissues^[3]^. Current treatments, such as non-surgical periodontal therapy (NSPT), access flap surgery and guided tissue regeneration (GTR), focus on disease control through removal of plaque biofilm deposits, and, where possible, GTR^[4,5]^. However, despite advancements in periodontal treatment, the complete restoration and regeneration of the functional periodontium, including alveolar bone, cementum and periodontal ligament (PDL) with functional attachment of Sharpey’s fibers is still largely unpredictable^[6-8]^. This clinical challenge is attributed to the complex, multi-tissue nature of the periodontal apparatus and the difficulty in achieving coordinated regeneration of these distinct but interconnected tissues. Traditional dental MSC-based therapies use living cells for repair, whilst cell-free approaches, such as small extracellular vesicles (sEVs), may offer a more effective alternative by promoting tissue regeneration with significantly lower risks of immune rejection, tumorigenicity, and engraftment^[9]^. Therefore, exploring the natural secretome [i.e., extracellular vesicles (EVs)] from dental MSCs and immune cells (monocytes or macrophages) is crucial for developing alternative ‘cell-free’ approaches in periodontal regenerative medicine, particularly when combined with advanced biomaterial delivery systems.

**Figure 1:**
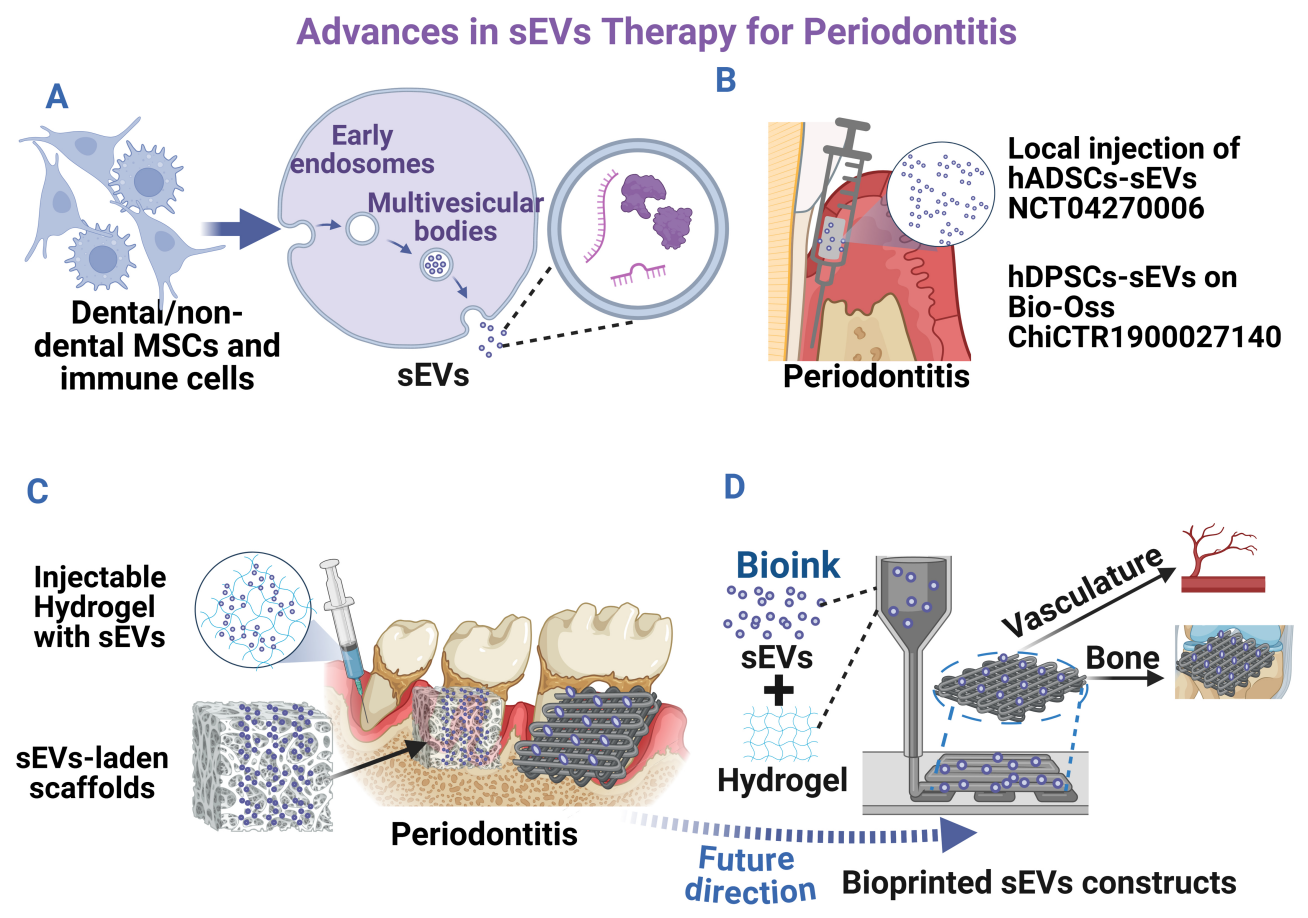
Advances in sEVs therapy for periodontitis. (A) sEVs biogenesis and secretion process; (B) Current clinical trial registry for treating periodontitis; (C) Current *in vivo* periodontal regeneration using sEV-laden scaffolds; (D) Bioprinted sEVs constructs for bone/periodontal regeneration. Created in BioRender. Zhang, C. (2025) https://BioRender.com/0yi01jv. sEVs: Small extracellular vesicles; MSCs: mesenchymal stem cells; hADSCs: human adipose-derived MSCs; hDPSCs: human dental pulp stem cells.

sEVs are membrane-enclosed nanoparticles (typically 30-200 nm) produced through the endosomal biogenesis pathway [Figure 1A] and secreted by diverse types of cells^[10]^, carrying microRNAs (miRNAs), proteins, lipids, and other molecules that reflect their parent cells and mediate intercellular communication^[10-12]^. Compared with living cells or soluble growth factors, both naïve and engineered sEVs offer advantages such as enhanced stability, reduced immunogenicity, and protection of their biomolecule cargo by a lipid bilayer^[12,13]^. In periodontal regeneration, sEVs derived from periodontal stem cells, including periodontal ligament cells (PDLCs), gingival MSCs (GMSCs), and dental pulp stem cells (DPSCs), showed promise in promoting osteogenic and cementogenic differentiation, angiogenesis, and macrophage polarization toward anti-inflammatory phenotypes^[14-17]^. For example, sEVs from apical papilla stem cells (SCAP-sEVs) can suppress pro-inflammatory cytokines [e.g., tumor necrosis factor α (TNF-α)] while decreasing the distance between the cementoenamel junction and alveolar bone crest (CEJ-ABC, a key indicator of clinical attachment loss) in a mouse periodontitis model^[18]^. In addition, sEVs from periodontal ligament stem cells (PDLSC-sEVs) are proven to be effective in promoting bone regeneration in rats with experimental periodontitis^[19]^, making them a promising adjunct to conventional periodontitis treatment. Dental follicle stem cell (DFSC)-derived sEVs, have also been reported to facilitate the alignment of PDL fibers with firm attachment to new cementum and alveolar bone, thus contributing to the reconstruction of periodontal tissues^[20]^. Notably, clinical translation is advancing [Figure 1B], with sEVs from autologous adipose MSCs administered via local injection [human adipose-derived MSCs (hADSCs)-sEVs; NCT04270006] and from autologous DPSCs incorporated into the biomaterial Bio-Oss [hDPSCs-sEVs; World Health Organization (WHO) ChiCTR1900027140] currently registered for phase I clinical trials in humans, although results remain pending. These developments suggest that dental MSCs-sEVs hold significant potential as safe and effective therapeutic agents for periodontal regenerative medicine.

The periodontal defect presents unique challenges, including bacterial contamination, inflammatory conditions, and the need for multi-tissue regeneration with specific spatial organization^[21]^. Despite their therapeutic potential, sEVs face challenges in achieving targeted delivery and sustained release within the complex periodontal disease microenvironment. To address these delivery challenges, various biomaterials [Figure 1C, e.g., collagen sponges^[22]^, β-tricalcium phosphate bioceramic (β-TCP)^[19]^] have been employed as carriers to sustain dental MSC-derived sEVs release before application to the periodontal microenvironment (reviewed in^[23]^). Although dental MSC-sEV-laden biomaterials have been extensively reviewed in *in vivo* bone regeneration^[24-30]^, their role in periodontal regeneration remains limited, as summarized in our review. However, conventional biomaterials often cannot precisely control sEVs distribution, release kinetics, or create the complex microenvironment necessary to recapitulate natural periodontal tissue regeneration. The periodontal apparatus requires coordinated regeneration of multiple tissues with distinct properties and spatial relationships, which traditional biomaterial approaches struggle to address effectively.

Recent advances in 3D bioprinting technology offer promising solutions to address the limitations of conventional biomaterial approaches. As one of the leading additive manufacturing techniques, 3D bioprinting uses computer-aided design (CAD) to deposit bioinks [e.g., gelatin, collagen, hyaluronic acid (HA)] and living cells in precise, layer-specific patterns, allowing the replication of natural tissue architecture and fabrication of specialized 3D constructs with tailored mechanical properties optimized for periodontal regenerative defects [Figure 1D]^[31]^. Various techniques, such as extrusion-based, inkjet, and laser-based bioprinting, can be applied in different contexts.

In bone tissue engineering, researchers have begun exploring the combination of sEVs therapy with 3D bioprinting to create spatially organized scaffolds for more precise tissue regeneration. When applied to periodontal regeneration, we propose that 3D bioprinted scaffolds loaded with sEVs may offer a novel approach to mimic the complex, multi-layered periodontal structures. These constructs can facilitate inflammation suppression, stimulate vascularization, and promote coordinated bone and cementum formation while supporting PDL regeneration. The integration of sEVs with 3D bioprinting presents a promising strategy to address the limitations of conventional therapeutic approaches, enabling precise and potentially patient-specific functional tissue regeneration^[32,33]^. The spatial control afforded by 3D bioprinting allows for the strategic placement of different sEVs populations within specific regions of the construct, potentially enabling zone-specific regeneration that mimics the natural periodontal architecture. For instance, osteogenic sEVs could be concentrated in regions intended for alveolar bone regeneration, while cementogenic sEVs could be localized to areas requiring cementum formation, and angiogenic sEVs could be distributed throughout to support vascularization.

While a number of studies have used bioprinted sEVs constructs for tissue regeneration^[12,16,34-40]^, most have focused on bone repair. Recent reviews provided insights into bioprinted sEVs constructs in tissue engineering and regenerative medicine^[31,33,41]^; however, limited reviews specifically address periodontitis and the regeneration of cementum and PDL, which are core components of the periodontal attachment apparatus. The PDL, in particular, requires regeneration of a highly specialized connective tissue with specific fiber orientation, cellular composition, and functional properties that differ significantly from other mesenchymal tissues. Similarly, cementum regeneration involves the formation of a mineralized tissue with distinct properties from bone, requiring specific cellular and molecular signals that may differ from those promoting osteogenesis. This review aims to summarize current research on bioprinted sEVs constructs in bone and periodontal regeneration, critically analyze the existing evidence, and discuss the limitations and future clinical translation of 3D bioprinted sEVs constructs in periodontal disease management. By identifying knowledge gaps and technological challenges, it provides a roadmap for advancing this promising therapeutic approach from bench to bedside, ultimately contributing to improved outcomes for patients with periodontal disease.

## CURRENT CLINICAL TRANSLATION OF sEVs FOR PERIODONTAL REGENERATION

The goal of functional periodontal regeneration is to reconstruct oriented PDL fibers (Sharpey’s fibers) firmly attached to both cementum and alveolar bone tissue, which remains a major challenge in the clinic. Also, periodontium has a limited capacity for regeneration, while it relies more on the MSC availability. Several types of MSCs, including PDLCs, GMSCs, and DPSCs, serve as renewable progenitor cells with the capacity to differentiate into PDL, cementum, and bone-forming cells. These MSCs can be recruited to the lesion site, where they contribute to tissue formation^[42]^. Osteoblasts and osteoclasts, the two main types of bone cells, are active during each stage of bone remodeling^[43]^ and play critical roles in alveolar bone regeneration as well. Latest evidence suggests that osteocytes are involved in initiating physiological bone remodeling in periodontitis, as well as assisting in inflammation-related changes^[44]^. Besides, bone homeostasis is highly regulated by the crosstalk between all kinds of bone cells and the surrounding microenvironment, and the roles of sEVs derived from these cells have been comprehensively reviewed^[45-48]^. Compared with bone cells such as osteoblasts, osteoclasts, and osteocytes, many experiments are conducted using more accessible stem cells with self-renewal and multidirectional differentiation capacities, which may have greater potential to comprehensively promote PDL and cementum regeneration in the complex periodontal microenvironment^[49]^. Also, immune cells such as T cells, monocytes, and macrophages are closely involved in the inflammatory periodontal diseases that serve as potential sources of sEVs^[48,50]^.

EVs have emerged as important mediators of intercellular communication within the cellular microenvironment, facilitating signal transduction and homeostatic regulation. As the smallest subtype of EVs, sEVs (typically 30-200 nm in diameter) can be detected in all biological fluids^[51]^. Therefore, in the periodontal microenvironment, saliva, blood, and gingival crevicular fluid can act as a natural medium for signal exchange between donor and recipient cells, highlighting the therapeutic potential of sEVs in periodontal treatment.

Recent reviews have summarized the regenerative function of sEVs derived from dental and non-dental MSCs, including PDLSCs, DPSCs, GMSCs and bone marrow MSCs (BMMSCs)^[17,42,52,53]^. These small vesicles have exhibited the capacity to promote bone tissue regeneration^[54]^, enhance angiogenesis^[55,56]^ and regulate inflammation^[57]^, making them promising therapeutic agents in periodontal treatment. However, only limited studies have used osteoblast-derived sEVs for periodontal regeneration, although sEVs from the MC3T3 cell line (osteoblast precursor cell line derived from mouse calvaria) and primary osteoblasts have already been shown to promote osteogenesis in *in vivo* defect models^[58,59]^. Despite the promising preclinical evidence for sEVs in periodontal regeneration, translation to clinical applications faces significant challenges. This section examines current clinical trials investigating sEV-based periodontal therapies and explores how biomaterial delivery systems address key translational barriers, including retention, stability, and controlled release [Figure 1B and C].

### Current clinical trials of sEVs in periodontal treatment

The clinical potential of sEVs for periodontal regeneration is currently being evaluated in several ongoing trials, although the results remain pending. A search conducted on the ClinicalTrials.gov database and the WHO registry platform identified two interventional studies exploring therapeutic applications of autologous MSC-derived sEVs.

The first trial (ChiCTR1900027140) investigates a combination therapy using DPSC-derived sEVs (DPSC-sEVs) integrated with DPSCs and bone substitute materials for GTR in periodontitis patients. This approach aims to evaluate both the safety and efficacy of the combined treatment modality. In contrast, the second trial (NCT04270006) employs a more direct approach, administering autologous adipose stem cell-derived sEVs (ADSC-sEVs) via local injection into periodontal pockets as adjunctive therapy to conventional scaling and root planing. It is noted that no results from these two clinical trial registries have been reported.

While these studies highlight the clinical potential of MSC-derived sEVs as regenerative therapies, they also reveal a critical limitation: neither local injection nor direct implantation can guarantee long-term retention of sEVs at the treatment site. This challenge highlights the need for advanced delivery systems that can overcome the inherent limitations of sEVs therapy.

### Biomaterial-enhanced sEVs delivery systems for periodontal treatment

Although the lipid bilayer structure of sEVs provides inherent protection for their bioactive cargo and enables stable cell-to-cell communication, several limitations hinder their clinical application. Local injection of sEVs into periodontal defects presents challenges related to rapid clearance, short half-life, off-target effects, and inadequate retention at the treatment site^[24,29,31,43,60]^. To address these limitations, researchers have developed composite systems that encapsulate sEVs within highly porous scaffolds or hydrogels, enabling sustained and prolonged retention while optimizing the synergistic benefits of both sEVs and biomaterial carriers.

**Figure 2:**
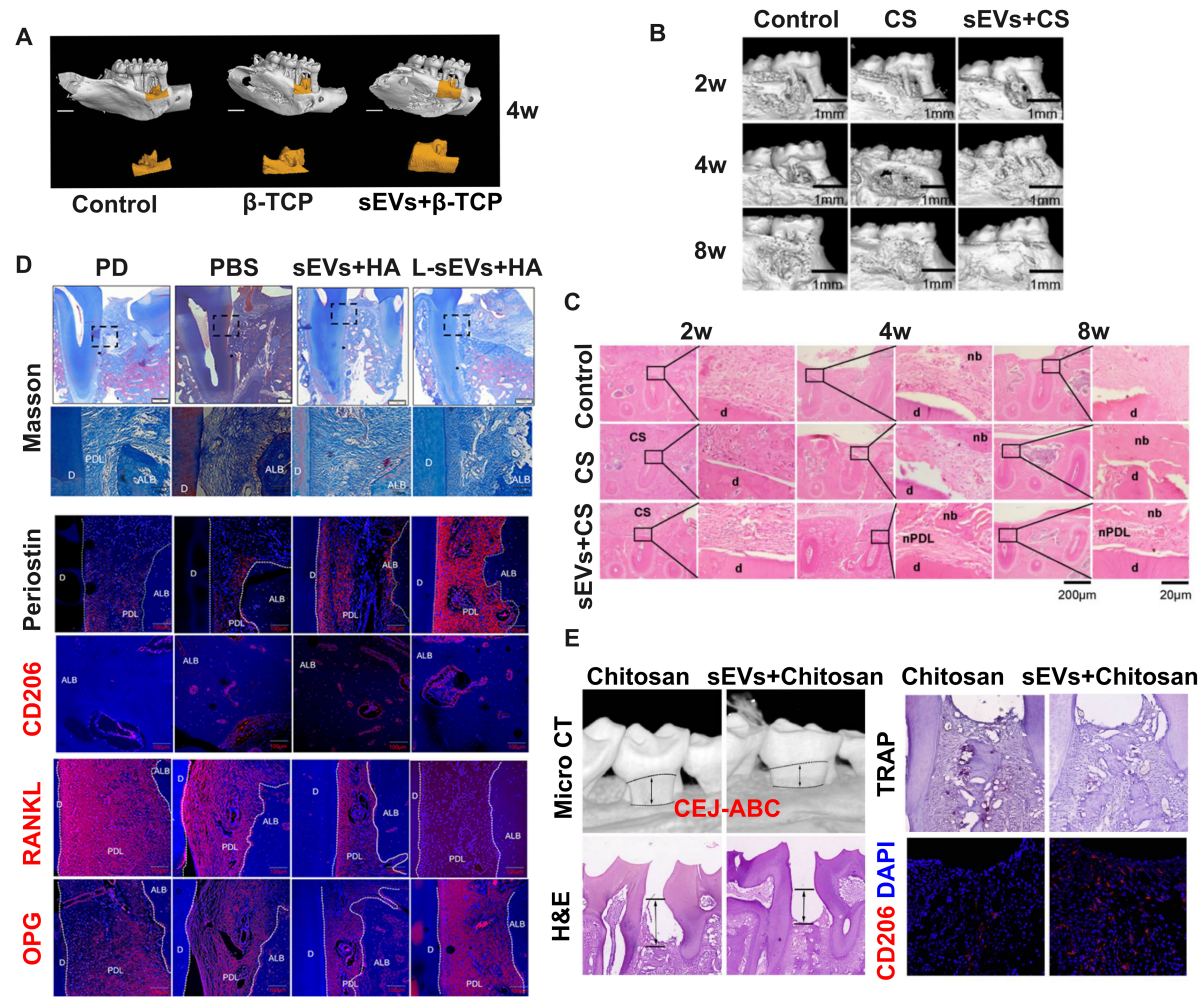
MSCS-sEV-laden biomaterials enhance periodontal regeneration in preclinical models. (A) Micro-CT reconstruction images demonstrating new bone formation in rat periodontal defects 4 weeks after treatment with PDLSC-sEVs loaded β-TCP scaffolds, compared to control treatments. Adapted from Ref.^[19]^; (B) Longitudinal micro-CT analysis showing progressive new bone formation at 2, 4, and 8 weeks post-treatment in control, CS, and DFSC-sEVs + CS groups. Scale bars = 1 mm. Adapted from Ref.^[20]^; (C) Histomorphological analysis using H&E staining reveals nb and nPDL formation in DFSC-sEVs + CS-treated defects. Note the organized tissue architecture compared to controls. Scale bars = 20 and 200 μm. Adapted from Ref.^[20]^; (D) Comparative histological analysis using Masson’s trichrome staining and immunofluorescence for Periostin, CD206, RANKL, and OPG in periodontal defects. Treatment with DFSC and L-DFSC-sEVs + HA increased periodontal ligament width, vessel density, and expression of regenerative markers (Periostin, CD206, a typical marker of anti-inflammatory M2 macrophage) while decreasing the RANKL/OPG ratio compared to controls. Scale bars = 100 μm. Adapted from Ref.^[67]^; (E) Therapeutic efficacy of DPSC-sEVs-loaded chitosan hydrogels in experimental periodontitis. Micro-CT reconstruction, H&E staining, TRAP staining, and CD206 immunofluorescence demonstrate that DPSC-sEVs + chitosan treatment reduces the CEJ-ABC distance and osteoclast numbers while promoting anti-inflammatory macrophage polarization (increased CD206 expression) compared to chitosan-only controls. Scale bars = 200 μm (micro-CT) and 100 μm (histology). Adapted from Ref.^[63]^. MSCs: Mesenchymal stem cells; sEVs: small extracellular vesicles; Micro-CT: micro-computed tomography; PDLSC-sEVs: periodontal ligament stem cell-derived small extracellular vesicles; β-TCP: beta-tricalcium phosphate; CS: collagen sponge; DFSC-sEVs: dental follicle stem cell-derived small extracellular vesicles; H&E: hematoxylin and eosin; nb: new bone; nPDL: new periodontal ligament; d: dentin; DFSC-sEVs + HA: DFSC-sEVs loaded in hyaluronic acid gel; L-DFSC-sEVs + HA: lipopolysaccharide-preconditioned DFSC-sEVs loaded in hyaluronic acid gel; ALB: alveolar bone; DPSC-sEVs: dental pulp stem cell-derived small extracellular vesicles; CEJ-ABC: distance between cementoenamel junction and alveolar bone crest; TRAP: tartrate-resistant acid phosphatase; OPG: osteoprotegerin; RANKL: receptor activator of nuclear factor kappa-B ligand; M2: M2 macrophage phenotype.

We have included selected current research^[19,20,22,61-70]^ that has focused on three primary biomaterial platforms for sEVs delivery: hydrogels, collagen sponges, and bone substitutes such as β-TCP [Table 1 and Figure 2]. Hydrogels and collagen sponges offer advantages of biocompatibility, biodegradability, and relative inertness compared to ceramic materials^[22]^, while β-TCP provides additional benefits through its inherent osteoconductivity and osteoinductivity^[69]^.

**Table 1:** Biomaterials as delivery carriers for dental and non-dental MSCs-sEVs in periodontal treatment

**sEVs**	**Biomaterial components**	**Main *in vivo* findings**	**Ref.**
40 µg MSCs-sEVs/Scaffold	Collagen sponge	Promote the regeneration of critical-size periodontal defect with haphazard fibers alignment in rats	[22]
150 μg/μL hPDLSC-sEVs	Matrigel/β-TCP powder	Increased the volume and density of alveolar bone with more OCN/RUNX2 positive cells in rats’ periodontal bone defect	[19]
1 µg/µL (sEVs/hydrogel) hPDLSC-sEVs	Matrigel	More new bone and mature collagen-1 fibers observed in rat periodontal bone defect with miR-200b/c over-expressed functionalized sEVs laden matrigel	[61]
1 μg/μL hPDLSC-sEVs	ADA-GEL hydrogel	Promoted new bone and bone-like tissue formation and increased BV/TV parameter in rat alveolar bone defect	[62]
50 μg hDPSC-sEVs/Scaffold	1% Chitosan hydrogel	Decreased the distance between the CEJ-ABC 4 weeks post-injection, rescuing alveolar bone loss caused by experimental periodontitis in mice	[63]
500 μg/mL DFSC-sEVs	1.25% gelatin and 2.5% laponite	Less CEJ-ABC distance, less inflammatory cells infiltration, well-oriented fibers and reduced bone loss in experimental periodontitis rats	[64]
DFSC-sEVs, dose/concentration not mentioned	Collagen sponge	New bone and periodontal ligament-like tissue formation observed in rats’ periodontal defect model after 4 weeks	[20]
100 μg DFSC-sEVs	Matrix collagen membrane	Exhibited a higher presence of fibrous tissues and new bone formation compared with the control group 2 weeks post-surgery	[65]
40 μg DFSC-sEVs	Collagen sponge	Promoted continuous trabecular bone formation, increased vessel numbers and OPN/MMP-2 expression in rats’ alveolar bone defects	[66]
4 μg/μL LPS-treated DFSC-sEVs (200 μg sEVs in 50 μL hydrogel)	5% hyaluronic acid gel	Enhance CD206 expression and new alveolar bone/PDL-like structures formation, promoted fibers attaching to cementum layer and lowered the ratio of RANKL/OPG expression after 8 weeks in dogs	[67]
500 μg/mL BMSC-sEVs	1.25% gelatin and 2.5% laponite	Lower the numbers of tartrate-resistant acid phosphatase-positive cells and inhibiting the development of periodontitis in rats	[68]
100 μg SHED-sEVs/Defect	5mg β-TCP/Defect	Facilitated neovascularization and new bone formation in rats’ periodontal defect model	[69]
100 μg/mL SHED-sEVs	6 g/L high-molecular-weight sodium hyaluronate and 5 μg/mL Cu^2+^	Decreased CEJ-alveolar ridge distance, attenuated inflammatory infiltration, and increased volume/total volume ratio (BV/TV) in mice periodontitis model	[70]

MSC: Mesenchymal stem cells; sEVs: Small extracellular vesicles; PDLSC: periodontal ligament stem cells; β-TCP: β-tricalcium phosphate bioceramic; DPSC: dental pulp stem cells; DFSC: dental follicle stem cells; BMSC: bone marrow mesenchymal stem cells; SHED**:** stem cells from human exfoliated deciduous teeth; OCN: Osteocalcin; RUNX2: runt-related transcription factor 2; ADA-GEL: alginate-gelatin crosslinked; BV: bone volume; TV: total volume; CEJ-ABC: distance between cementoenamel junction and alveolar bone crest; MMP-2: matrix metalloproteinase-2; LPS: lipopolysaccharide; CD206: cluster of Differentiation 206; PDL: periodontal ligament; RANKL: receptor activator of nuclear factor kappa-B ligand; OPG: osteoprotegerin.

PDLSCs represent one of the most promising cell sources for periodontal regenerative medicine due to their accessibility and multipotent differentiation capacity. PDLSC-sEVs have demonstrated particular efficacy in promoting both bone formation and vascularization processes. Lei *et al*. pioneered their application by incorporating them into a matrigel/β-TCP composite scaffold at a concentration of 150 μg/μL and implanting the construct into rat periodontal bone defects^[19]^. Four-week follow-up using microcomputed tomography (micro-CT) and histological analysis revealed increased alveolar bone volume and density [Figure 2A], with enhanced expression of osteogenic markers (Osteocalcin and Runt-related transcription factor 2), demonstrating the synergistic potential of PDLSC-sEVs and biomaterial scaffolds in bone regeneration^[19]^. Building on these findings, the same research group further enhanced therapeutic efficacy through genetic engineering approaches. By overexpressing mechanically responsive miRNA-200b/c in PDLSCs and isolating the resulting sEVs, they developed an injectable Matrigel system with superior performance in rat periodontal defects^[61]^. This approach demonstrates the potential for engineering sEVs to optimize their regenerative capacity.

Alternative scaffold designs have also shown promise. Researchers using alginate-gelatin crosslinked hydrogels (ADA-GEL) as PDLSC-sEVs carriers reported enhanced new bone formation compared to control and hydrogel-only groups, with increased bone volume/tissue volume ratios and more extensive bone-like tissue formation detected by Masson staining^[62]^.

DFSCs, typically harvested from immature third molars and premolars in young patients, share developmental and functional similarities with PDLSCs^[71]^. Multiple studies have demonstrated successful integration of DFSC-sEVs with biomaterials such as collagen sponges and hyaluronic acid gel, showing sustained retention at 2 and 4 weeks post-transplantation [confirmed by the red fluorescent dye PKH26 (PKH26 Red Fluorescent Cell Linker Kit for General Cell Membrane Labeling; Sigma-Aldrich) and the green fluorescent dye DiO (3,3’-dioctadecyloxacarbocyanine perchlorate), both of which are lipophilic tracers for labeling membranes] and promoting diffuse bone formation and PDL-like tissue regeneration [Figure 2B-D]^[20,66,67]^.

Innovation in hydrogel design has further advanced DFSC-sEVs delivery. Shi *et al*. developed a laponite-gelatin hydrogel system for DFSC-sEVs delivery, demonstrating sustained release profiles and a significant reduction in experimental periodontitis-induced bone loss^[64]^. Treatment outcomes included decreased distance between the CEJ-ABC, reduced inflammatory cell infiltration, and improved fiber orientation in periodontal tissues, suggesting the effectiveness of DFSC-sEVs-loaded laponite-gelatin hydrogel in periodontal regeneration.

Other MSC sources have also contributed to the expanding evidence base. DPSC-sEVs incorporated into chitosan hydrogels showed protective effects against alveolar bone loss [e.g., bone tissue regeneration revealed by decreased CEJ-ABC distance, and lower osteoclast activity revealed by fewer tartrate-resistant acid phosphatase (TRAP+) cells] in experimental periodontitis models [Figure 2 E]^[63]^. Similarly, sEVs from stem cells of human exfoliated deciduous teeth (SHED) demonstrated osteogenic potential when combined with β-TCP scaffolds^[69]^ or HA hydrogels supplemented with Cu^2+^^[70]^, contributing to both neovascularization and anti-inflammatory activities. BMSCs have provided additional validation for the biomaterial-sEVs approach. BMSC-sEVs integrated into hydrogel systems effectively reduced TRAP+-positive cell numbers and inhibited periodontitis progression through regulation of the Osteoprotegerin/Receptor activator of nuclear factor kappa-B ligand/Receptor activator of nuclear factor kappa-B (OPG-RANKL-RANK) signaling pathway and macrophage polarization modulation^[68]^. Chew *et al*. loaded sEVs from MSCs on a collagen sponge, which was proven to promote regeneration of critical-size periodontal defects with haphazard fiber alignment^[22]^. Shen *et al*. incorporated DPSC-sEVs with chitosan hydrogel, and then the mixture was injected locally into the periodontal defect around the maxillary left second molar^[63]^. Micro-CT and histology analysis showed that CEJ-ABC decreased 4 weeks after injection, rescuing alveolar bone loss resulting from experimental periodontitis.

While existing studies demonstrate the potential of sEVs-biomaterial composite systems for periodontal regeneration, several critical challenges remain unresolved. Key issues include standardization of sEVs quantity and quality for consistent therapeutic efficacy, optimization of scaffold composition and manufacturing processes, and development of delivery systems that address the unique architectural requirements of periodontal tissues.

The complex multiphasic structure of the periodontium, encompassing both soft tissues (gingiva and PDL) and hard tissues (cementum and alveolar bone), necessitates sophisticated scaffold designs capable of supporting differential tissue regeneration^[6,7]^. Current biomaterial approaches, while promising, lack the precision required to recapitulate the spatially organized architecture of native periodontal tissues. These limitations have sparked interest in advanced manufacturing technologies, particularly 3D bioprinting, which offers the capability to replicate natural extracellular matrix (ECM) structures and create precisely controlled layer-by-layer 3D architectures^[40,72]^. This technological advancement represents the next frontier in sEV-based periodontal regenerative medicine.

## BIOPRINTING TECHNOLOGY FOR sEVs-ENHANCED PERIODONTAL REGENERATION

Three-dimensional bioprinting represents a state-of-the-art manufacturing technology using bioinks containing biomaterials, living cells^[73]^ and bioactive molecules^[12,74]^ to fabricate scaffolds with precisely controlled architecture, porosity, and mechanical properties that closely mimic native tissue characteristics^[75]^. While traditional bioprinting approaches incorporate living cells into these constructs, this strategy presents significant challenges, including storage limitations, compromised cell viability, and potential immune rejection^[13,76,77]^, which may hinder their clinical translation. To address these limitations, cell-free strategies - particularly the incorporation of sEVs and growth factors - have emerged as promising alternatives for enhancing regenerative potential while circumventing cell-related complications. However, optimizing printing parameters, bioink formulations, and clinical-grade sEVs production remains a critical challenge for successful translation.

### Advantages of 3D bioprinted EVs constructs

Despite their therapeutic promise, systemically administered sEVs face rapid clearance from circulation, often within minutes of injection^[12]^, significantly limiting their efficacy^[34]^. While conventional biomaterials such as injectable hydrogels have favorable biocompatibility and performance to address this issue as a delivery platform^[78]^, achieving optimal porosity remains crucial to prevent premature burst release and ensure sustained therapeutic delivery^[79]^. Three-dimensional bioprinting offers distinct advantages over injectable hydrogel systems by enabling precise replication of natural ECM architecture and providing superior control over spatial and temporal sEVs patterning^[80]^. This technology allows fabrication of scaffolds with fine-tuned structural parameters^[81]^ and controlled porosity^[36]^ that enable sustained release while preserving sEVs biological activity. Unlike simple encapsulation methods, bioprinting permits the creation of complex, multi-layered constructs that can accommodate the diverse regenerative requirements of periodontal tissues.

**Figure 3:**
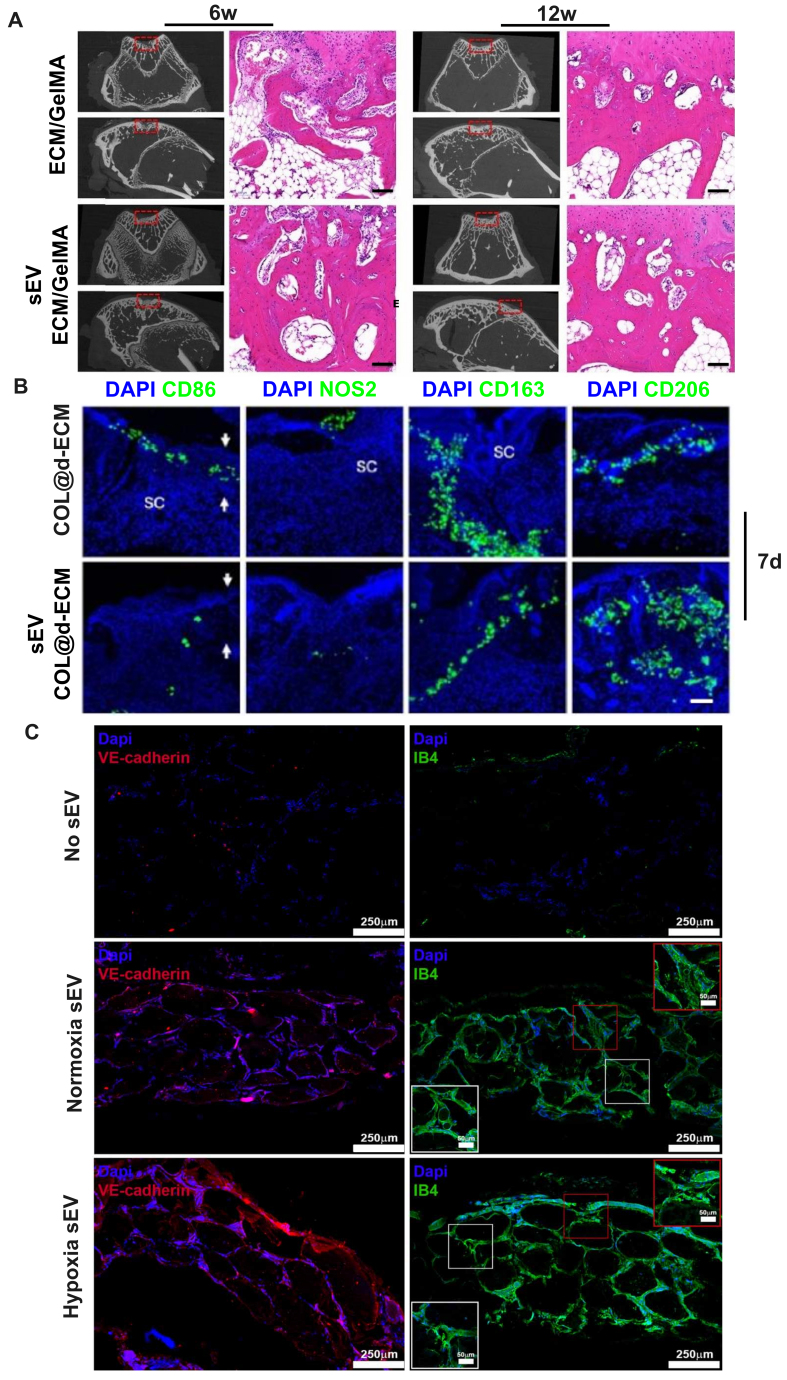
Bioprinted sEVs constructs promoted osteogenesis, immunomodulation and angiogenesis, key processes in bone regeneration. (A) Micro-CT reconstruction and histomorphological analysis of bioprinted GelMA + d-ECM + BMSCs-sEVs constructs in rabbit subchondral bone repair. Adopted from Ref.^[34]^. Scale bars = 100 μm; (B) Immunofluorescence staining of wound tissues showing pro-inflammatory (CD86, NOS2) and anti-inflammatory (CD163, CD206) markers at different time points in a rat subcutaneous wound healing model treated with bioprinted COL + d-ECM + M2-sEVs constructs. Adopted from Ref.^[38]^. Scale bar = 100 μm; (C) Bioengineered vessel structures stained with VE-cadherin (Red) and Isolectin B4 (Green), induced by bioprinted GelMA + alginate + HUVECs-sEVs constructs. Adopted from Ref.^[37]^. GelMA + d-ECM + BMSCs-sEVs: BMSCs-sEVs laden in GelMA and cartilage-derived d-ECM; COL + d-ECM + M2-sEVs: M2-sEVs laden in collagen type-1 and chicken skin-derived d-ECM; GelMA + alginate + HUVECs-sEVs: HUVECs-sEVs laden in GelMA and alginate; sEVs: small extracellular vesicles; GelMA: gelatin methacryloyl; d-ECM: decellularized extracellular matrix; BMSCs: bone marrow mesenchymal stem cells; COL: collagen type I; M2: M2 macrophage phenotype; VE-cadherin: vascular endothelial cadherin; HUVECs: human umbilical vein endothelial cells.

Recent investigations have demonstrated the regenerative potential of bioprinted sEVs constructs in bone regeneration through enhanced osteogenesis, angiogenesis, and immunomodulation [Table 2 and Figure 3]^[12,16,34-40]^. Those studies employed micro-CT reconstruction and histomorphological analysis for investigating the osteogenic potential of bioprinted sEVs constructs^[34]^, and applied immunofluorescence staining for revealing the inflammatory level [pro-inflammatory clusters of differentiation (CD)86, Nitric oxide synthase 2 markers, and anti-inflammatory CD163, CD206 markers]^[38]^ and bioengineered vessels structures (endothelial cells markers such as Vascular endothelial-cadherin and Isolectin B4)^[37]^. These findings indicate synergistic benefits and substantial potential for effective periodontal tissue regeneration. However, challenges in standardizing sEVs isolation, optimizing yield, and establishing characterization protocols continue to hinder the consistent fabrication of EV-integrated constructs^[31]^. To fully harness the potential of 3D bioprinted sEVs constructs in clinical applications, efforts are needed to optimize production, improve scaffold design, and establish robust quality control standards for consistency, safety, and therapeutic efficacy.

**Table 2:** Selected studies using 3D bioprinted sEVs constructs for bone, vasculature and periodontal regeneration

**Bioprinted sEVs**	**Bioink components**	**Key *in vitro* results**	**Main *in vivo* findings**	**Ref.**
Bioprinted sEVs constructs for bone and vasculature regeneration				
BMSCs-sEVs via stereolithography bioprinting	GelMA, cartilage-derived d-ECM and 200 μg/mL sEVs	Increased chondrocyte migration	Activated M2 macrophage polarization and promoted subchondral bone regeneration in rabbit osteochondral defect model	[34]
M0-eBMP2-sEVs via inkjet-based bioprinting	100 μg/mL sEVs loaded with BMP-2 in PBS with 10% glycerol, filtration by 0.22 μm membrane	Promoted C2C12 cells differentiation toward the osteogenic lineage	Induced localized heterotopic ossification in the mouse muscle pocket model	[35]
BC-M0-sEVs via microextrusion	10% Alginate, 5% HA and 200 μg/mL sEVs	Induced the immunomodulation of macrophages, osteogenic differentiation of BMSCs, and angiogenesis of HUVECs	N/A	[12]
hADSCs-sEVs via pneumatic-driven microextrusion	30 μg/mL sEVs, d-ECM, Gel, QCS and nHAp	Promoted proliferation and osteogenesis in hBMSCs Promoted migration and angiogenesis in HUVECs	Enhanced bone regeneration and vascularization in rat skull defect after 10 weeks Enhanced vascular formation after subcutaneous implantation in mice	[36]
HUVECs-sEVs via microextrusion	6% of GelMA and 4% of alginate, 4 × 10^9^ particles/mL sEVs, filtration by 0.22 μm membrane	Induced the formation of spindle-shaped multicellular structures in PBMCs	Enhanced extensive neovascularization in subcutaneous implantation model	[37]
M2-sEVs via pneumatic-driven microextrusion	1% COL and 10% chicken skin-derived d-ECM, dose/concentration of sEVs was unspecified	Increased the quantity of tube formation and CD31 gene expression in hBMSCs	Facilitated angiogenesis and M2 macrophage polarization in wound healing model	[38]
hUMSCs-sEVs via pneumatic-driven microextrusion	5% GelMA, 0.2% LAP, 100 μg/ml BP (upper layer) or 3% β-TCP (lower layer) and 500 μg/mL sEVs	Promoted chondrogenic and osteogenic differentiation of BMSCs	Facilitated articular cartilage and subchondral bone regeneration after 14 weeks	[39]
hADSCs-sEVs via pneumatic-driven microextrusion	9% GelMA, 2% OHA, 2% HA-DA, 2% DCM (upper layer) or DBM (lower layer) and 100 µg/mL sEVs	Promoted chondrogenic and osteogenic differentiation of BMSCs	Facilitated cartilage-bone integrated repair with continuous subchondral bone formation after 12 weeks	[40]
Bioprinted sEVs constructs for periodontal regeneration				
hGFs-sEVs and hPDLCs-sEVs via microextursion	10% GelMA and 10^10^ particles/mL of sEVs	Promoted cell adhesion, ligamentous, osteogenic, and cementogenic differentiation of hBFP-MSCs	N/A	[16]

sEVs: Small extracellular vesicles; BMSCs: bone marrow-derived mesenchymal stem cells; GelMA: gelatin methacrylate; d-ECM: decellularized extracellular matrix; BC-M0-sEVs: sEVs from M0 state RAW 264.7 cells induced by β-TCP; BMP-2: bone morphogenetic protein-2; PBS: phosphate-buffered saline; HA: hyaluronic acid; hADSCs: human adipose-derived mesenchymal stem cells; QCS: quaternized chitosan; nHAp: nano-hydroxyapatite; HUVECs: human umbilical vein endothelial cells; PBMCs: peripheral blood mononuclear cells; COL: collagen type-1; CD31: cluster of differentiation 31; hUMSCs: human umbilical cord mesenchymal stem cells; LAP: lithium phenyl-2,4,6-trimethylbenzoylphosphinate; BP: black phosphorus; β-TCP: β-tricalcium phosphate bioceramic; OHA: oxidative hyaluronic acid; HA-DA: dopamine-conjugated hyaluronic acid; DCM: d-ECM of cartilage; DBM: d-ECM of bone; hGFs: human gingival fibroblasts; hPDLCs: human periodontal ligament cells; hBFP-MSCs: human buccal fat pad-derived mesenchymal stem cells; N/A: not applicable.

### 3D bioprinting technique selection and parameters optimization

The selection of appropriate bioprinting technology is critical for preserving sEVs bioactivity during fabrication and implantation. Technology choice depends on both the properties of the target tissue and the strengths and limitations of each technology, as summarized in Table 3. Considering the potential risks associated with high temperatures and high-intensity ultraviolet (UV) irradiation^[75]^, gentle methods such as extrusion^[12,36-40]^, inkjet^[35]^ and laser-based technology^[34]^ are most widely employed to avoid compromising the bioactivity of encapsulated sEVs.

**Table 3:** Comparison of 3D bioprinting strategies

**Technology**	**Merits**	**Limitation**	**sEVs compatibility**
Extrusion-based	· Bioinks with various viscosities · Ease of use · Cost-effective	· Low resolution · Raise shear stress · Impair cell viability	Good - widely used for sEVs
Inkjet-based	· High speed and resolution · High cell density	· Impair cell viability · Not suitable for viscous bioinks	Moderate - requires optimization
Laser-based	· High resolution · High-viscosity bioinks · High cell density with low shearing	· Equipment complexity · High operational costs · Specialized materials needed	Excellent - gentle process

3D: Three-dimensional; sEVs: small extracellular vesicles.

Each method has its pros and cons based on technical principles. Extrusion-based bioprinting is widely used due to its ability to print high-viscosity bioinks and incorporate cells or sEVs in a controlled manner^[82]^. Inkjet bioprinting, on the other hand, provides high-resolution deposition but is limited by bioink viscosity and cell viability constraints^[83]^. Laser-assisted bioprinting offers precise spatial control and high resolution but requires specialized equipment^[84]^.

Beyond technology selection, bioink formulation must enable stable 3D construct formation with high fidelity while exhibiting mechanical properties that match target tissue characteristics^[60]^. The ideal bioink should support cell infiltration post-implantation, promoting directional differentiation and tissue-specific regeneration. Critically, the viscoelastic properties and mechanical strength of bioinks necessitate careful characterization and optimization^[85]^, which may be altered upon changing bioink components, such as sEVs incorporation. Maiullari *et al*. provided the only comprehensive analysis of rheological properties both with and without sEVs incorporation, demonstrating that viscosity remained largely unaffected at low shear rates, thereby ensuring bioink stability and printability^[37]^. This finding suggests that sEVs integration does not fundamentally compromise bioink performance, though systematic evaluation across different formulations remains needed.

Achieving optimal printability and structural integrity requires careful integration of bioprinting technology selection with bioink formulation optimization, emphasizing the need for systematic approaches to parameter optimization.

### Clinical-grade extracellular vesicle enrichment and characterization

The production of clinical-grade sEVs demands rigorous attention to every aspect of the manufacturing pipeline, from cell sourcing through purification and quality control, to meet stringent regulatory requirements. Key factors include cell source, EV yield, isolation method, characterization, and storage, all of which influence reproducibility and translational potential. A standardized workflow is essential for complying with regulatory standards to generate sEVs of good manufacturing practice (GMP) grade, with consistent miRNA and protein profiles for clinical use^[86,87]^.

#### Cell source selection and conditioning

Parent cell evaluation represents a critical initial step, as cell type and physiological state directly influence sEVs yield, purity, functionality, and clinical applicability^[75]^. For instance, sEVs derived from human PDLCs (hPDLCs) facilitate periodontal regeneration from ligamentous, osteogenic, and cementogenic perspectives^[16]^, while sEVs derived from human umbilical vein endothelial cells (HUVECs) enhanced extensive neovascularization correspondingly^[37]^. Furthermore, the physiological state of the cells should also be considered (i.e., hypoxia, inflammation and senescence), which can significantly influence the quality and functional consistency of sEVs and batch-to-batch variability^[43]^, hindering the scalability of production for clinical applications. Li *et al*. demonstrated that hypoxic preconditioning of DPSCs improved the angiogenic potential of derived sEVs, enhancing HUVEC proliferation, migration, and tube formation while altering the proteomic profile^[88]^. Similarly, inflammatory stimuli such as TNF-α and Interleukin 1α (IL-1α) can modulate sEVs miRNA and protein landscapes, thereby modulating the angiogenic potential of parent MSCs^[89]^. These conditioning approaches represent simple, cost-effective modification methods that do not affect the structural integrity of sEVs or require additional purification steps, making them suitable for large-scale production.

#### sEVs isolation and purification technologies

Beyond the cell source, the methods used for isolation, purification, and characterization are essential in the production of clinical-grade sEVs. While ultracentrifugation (UC) remains a widely used gold standard, new technologies such as tangential flow filtration (TFF), size-exclusion chromatography (SEC) and immunoaffinity have been developed for high efficiency, yield and purity^[15,75,90]^. Among them, TFF, a technique that couples permeable membrane filtration with controlled flow to achieve efficient sEVs concentration^[91]^, enables the gentle and efficient separation from large sample volumes^[92]^. SEC provides gentle separation with high efficiency and purity by eliminating protein contaminants based on size differences without affecting vesicle structure^[93]^. In addition, immunoaffinity-based isolation employs antibodies targeting sEVs surface markers (e.g., CD63, CD9, CD81), thereby increasing isolation specificity^[94]^. However, immuno-isolation may exclude certain subsets as EVs are heterogeneous and do not uniformly express the same surface markers. No studies included in our review enriched sEVs using this approach, and it remains unclear whether any sEVs subtype has superior regenerative potential. Currently, most bioprinting studies continue to employ UC^[34,36,37]^; however, combination approaches (e.g., FTT + SEC, UC + SEC) are increasingly being explored^[92,95]^. Given the complementary advantages of different isolation methods, combinatorial strategies may become standard practice to facilitate robust and reproducible sEVs research.

A key limitation in many studies is the quantification of sEVs based solely on total protein content rather than vesicle number. Such approaches overlook potential non-EV contaminants and exomere fractions, reducing reproducibility and comparability across studies. Although nanoparticle tracking analysis (NTA) is not ideal, it provides a more meaningful quantitative assessment of EV concentration and size distribution. Only two studies^[16,37]^ were conducted based on sEVs particle numbers, indicating the lack of standardized quantification methods in current EV research. As mentioned in the minimal information for studies of extracellular vesicles 2018 (MISEV2018) guideline, protein:particle, protein:lipid and RNA:particle ratios have been proposed as possible purity metrics^[90]^, which will be essential for better reproducibility and reliability.

#### sEVs characterization and quality control

Following the latest MISEV guideline^[96]^, comprehensive characterization of sEVs is essential to ensure product identity and quality, which mainly includes particle concentration and size distribution, morphological evaluation, protein composition, and surface marker profiling^[90]^. These characteristics are evaluated using complementary analytical techniques: NTA for particle quantification and sizing^[97]^, transmission electron microscopy (TEM) for morphological assessment, mass spectrometry and Western blotting for protein profiling^[98]^, and nanoflow cytometry for individual vesicle analysis and surface markers detection^[99]^. Also, each method has its limitations; for example, electron microscopy is only for morphology but not for quantification, which proves the necessity of combining multiple methods for a comprehensive characterization. Storage protocols are crucial for maintaining sEVs stability and bioactivity, with current consensus supporting storage at -80 °C^[100]^. However, optimization of storage protocols remains a challenge, especially considering the heterogeneity of sEVs subtypes and differing stability profiles^[101]^.

The isolation and characterization methods reported in the nine studies involving 3D-bioprinted sEVs constructs are summarized in Table 4. As shown, the methodologies varied significantly. UC was mostly utilized for isolation, and all studies employed more than three methods for thorough characterization.

**Table 4:** sEVs isolation and characterization methods in 3D bioprinted sEVs constructs

**Bioprinted sEVs**	**Isolation method**	**Characterization method**	**Ref.**
BMSCs-sEVs	300 × *g* for 15 min, 2,500 × *g* for 15 min, passed through a 0.22 μm Millipore filter, 4,000 × *g* to concentrate to 200 µL, 100,000 × *g* for 1 h and 4,000 × *g* to 200 µL	DLS, TEM, WB (TSG101, CD9, and CD63), confocal microscopy (DiO)	[34]
M0-eBMP2-sEVs	2,000 × *g* for 10 min at 4 °C, 10,000 × *g* for 30 min at 4 °C, passed through a 0.22 µm Millipore filter and mini-SEC using 1.5 cm × 12 cm mini-columns (Bio-Rad) packed with 10 mL of Sepharose 2B (Sigma-Aldrich) equilibrated with phosphate-buffered saline	WB (TSG101, CD9, and CD63), TEM, tunable pulse resistive sensing, FCS (PKH26), confocal microscopy (PKH26)	[35]
BC-M0-sEVs	300 × *g* for 10 min, 2,000 × *g* for 20 min, pass through 0.2 µm sterilized filter, then use Total Exosome Isolation Reagent (Invitrogen), 10,000 × g for 60 min, resuspend in PBS and stored at -80 °C	TEM, NTA, BCA, WB (CD9, CD81, TSG101)	[12]
hADSCs-sEVs	300 × *g* for 10 min, 2,000 × *g* for 20 min, 10,000 × *g* for 30 min, serially pass through 0.45 and 0.22 μm filter, 100,000 × *g* for 70 min for twice	TEM, NTA, BCA, FCS (CD105, CD73, CD90, CD34), zeta potential measurement, confocal microscopy (PKH26)	[36]
HUVECs-sEVs	500 × *g* for 15 min, 1,000 × *g* for 25 min, 125,000 × *g* for 90 min at 4 °C	NTA, WB (CD9, CD81), FCS (CD9, CD81 and CD63), ELISA (VEGF, PIGF, VEGFR1, VEGFR2), TEM, confocal microscopy	[37]
M2-sEVs	800×g, then use Total Exosome Isolation Kit (Thermo-Fischer Scientific)	NTA, TEM, WB (CD9, CD63), confocal microscopy (Deep Red CellMask plasma membrane stain)	[38]
hUMSCs-sEVs	Not mentioned	Not mentioned	[39]
hADSCs-sEVs	300 × *g* and 2,000 × *g* for 10 min, 10,000 × *g* for 30 min, pass through 0.22 µm Millipore filter, 100,000 × *g* for 2 h at 4 °C	NTA, TEM, WB (CD81, ALIX and TSG101)	[40]
hGFs-sEVs/ hPDLCs-sEVs	300 × *g* for 15 min, 2,600 × *g* for 15 min, 16,000 × *g* for 20 min, pass through SEC columns and collect 7-15 fractions (100 μL), concentrate to 500 μL by 14,000 × *g* for 5 min	TEM, cryo-EM, FTIR, NTA, BCA, ELISA (CD9), confocal microscopy (DiO), MACSPlex Exosome Kit (for 37 EV surface markers)	[16]

sEVs: Small extracellular vesicles; BMSCs: bone marrow-derived mesenchymal stem cells; M0: naïve macrophages; BMP2: bone morphogenetic protein 2; BC-M0-sEVs: small extracellular vesicles from M0-state RAW 264.7 cells induced by β-tricalcium phosphate bioceramic (β-TCP); hADSCs: human adipose-derived stem cells; HUVECs: human umbilical vein endothelial cells; M2: alternatively activated macrophages; hUMSCs: human umbilical mesenchymal stem cells; hGFs: human gingival fibroblasts; hPDLCs: human periodontal ligament cells; DLS: dynamic light scattering; TEM: transmission electron microscopy; WB: western blot; FCS: flow cytometry; NTA: nanoparticle tracking analysis; BCA: bicinchoninic acid assay; ELISA: enzyme-linked immunosorbent assay; SEC: size-exclusion chromatography; Cryo-EM: cryo-electron microscopy; FTIR: Fourier-transform infrared spectroscopy; MACSPlex: MACSPlex Exosome Kit.

### Bioink formulation and optimization strategies

Among the nine studies investigating bioprinted sEVs constructs, bioink compositions demonstrate considerable diversity, reflecting the need to match material properties with target tissue requirements and research objectives. Periodontal regeneration presents unique challenges beyond the osteogenesis, angiogenesis, and immunomodulation processes common to bone regeneration^[102]^, as successful outcomes require coordinated cementum formation and aligned PDL fibre regeneration^[6,8]^, demanding more bioactive scaffolds.

In the included studies, only one comprehensively evaluated the effects of 3D bioprinted M0-sEVs from three key perspectives^[12]^: osteogenesis, angiogenesis, and immunomodulation. The authors employed β-TCP-induced M0-state RAW 264.7 cells-derived sEVs (BC-M0-sEVs, 200 μg/mL), 10% alginate and 5% HA as bioinks for 3D bioprinting. The resulting scaffolds demonstrated excellent printability across various geometric configurations while maintaining sufficient elasticity and strength to withstand mechanical deformation. Notably, sEVs functioned as nanoparticle fillers that enhanced hydrogel network density and scaffold modulus, achieving high retention rates and sustained release over one month. The bioink selection considered not only alginate-HA hydrogel printability but also the effects of bioceramic ion products (SiO_4_^4-^, Ca^2+^, Mg^2+^) on sEVs secretion to enhance osteogenic performance. The bioprinted constructs successfully promoted macrophage polarization from pro-inflammatory to anti-inflammatory phenotypes, enhanced BMSC osteogenic differentiation, and supported HUVEC migration and tube formation.

Gelatin Methacrylate (GelMA) represents the most commonly employed bioink component across included studies^[16,34,37,39,40]^, closely resembling essential ECM properties for cellular support and tissue formation while offering tunable biological functions and adjustable physical characteristics^[103,104]^. For example, Maiullari *et al*. used GelMA, alginate, and 4 × 10^9^ particles/mL HUVEC-sEVs, observing spindle-shaped multicellular structure formation and extensive neovascularization *in vitro* and *in vivo*, respectively, validating the angiogenic efficacy of bioprinted constructs^[37]^ [Figure 3C]. Decellularized ECM (d-ECM) biomaterials, which were also widely used among included studies^[34,36,38,40]^, offer unique advantages for bioprinting applications due to their inherent biocompatibility and bioactivity, enabling engineering of porous scaffolds that recapitulate native tissue structural aspects^[105]^. Unlike GelMA, d-ECM processing involves cellular component removal while preserving complex 3D ultrastructural and chemical signaling cues. Recent evidence indicates that ECM from different tissue regions possesses distinct properties, and d-ECM biomaterials inherit these characteristics^[106]^. Li *et al*. applied d-ECM derived from bone and cartilage tissues, respectively, combined with 100 μg/mL hADSC-derived sEVs to fabricate double-network hydrogel scaffolds targeting different tissue types^[40]^. This approach promoted both chondrogenic and osteogenic BMSC differentiation, enabling concurrent cartilage and subchondral bone regeneration. Given the distinct biomechanical properties of various periodontal tissues, d-ECM may offer advantages for creating tissue-specific, multiphasic scaffolds that accurately mimic periodontal tissue composition and organization.

#### Bioceramics and growth factors

External factors, including bioceramics, growth factors, and polysaccharides, can be incorporated into bioinks according to research objectives and target tissue requirements. Kang *et al*. added quaternized chitosan (QCS) and nano-hydroxyapatite (nHAp) to gelatin/d-ECM bioinks containing 30 μg/mL hADSC-derived sEVs^[36]^. QCS provided enhanced antibacterial activity^[107]^, while nHAp increased surface roughness for improved cell adhesion and induced osteoblast osteogenic differentiation through appropriate cell-material interactions^[108]^.

#### Engineered sEVs

Advanced approaches involve pre-loading therapeutic molecules into sEVs before bioink incorporation. In one study, bone morphogenetic protein-2 (BMP-2) was loaded into sEVs derived from murine J774A.1 monocytic cells (uninduced M0 state) before bioprinting^[35]^. These engineered vesicles (eBMP2-sEVs) enhanced C2C12 (immortalized mouse myoblast cell line) osteogenic differentiation *in vitro* and induced significant heterotopic ossification *in vivo*^[35]^.

While hydrogels represent ideal carrier platforms for bioactive factors, bioink stability following component addition requires careful consideration. Researchers found that nHAp could enhance bioink stability and prevent collapse post-extrusion; however, excessive nHAp concentrations impaired stability as storage modulus (G’) and loss modulus (G’’) decreased with reduced relative gel concentration^[36]^. For hydrogel systems, maintaining low swelling rates is essential to prevent large deformations under mechanical stress, which is vital for bone tissue repair applications. Sun *et al*. incorporated β-TCP into hydrogels to improve osteoconductivity, finding that increasing β-TCP concentration reduced swelling while simultaneously increasing compressive strength^[39]^. After comprehensive parameter evaluation, they selected 3% as the optimal β-TCP concentration.

These findings emphasize that scaffold properties must be thoroughly characterized using scanning electron microscopy, mechanical testing, swelling analysis, degradation assessment, and other relevant evaluations following bioprinting to ensure optimal performance for intended applications.

### Crosslinking methods of 3D bioprinting process

Bioinks with certain characteristics, such as good biocompatibility, stability, and favorable mechanical and rheological properties, can be transformed into 3D constructs via a crosslinking process [Table 5]. Crosslinking is a crucial step in 3D bioprinting, allowing the achievement of desirable biomechanical stability of printed structures. The main methods of chemical, physical, or enzymatic crosslinking used in 3D bioprinting studies have been reviewed^[109]^, in order to develop biomimetic and sustainable 3D constructs and improve constructs’ performance. Among the 3D bioprinting technologies, extrusion-based printing has been proven to have greater impacts on the rheological behaviour of bioinks^[110]^; thus, the effect of crosslinking reactions is more significant in extrusion-based bioprinting methods^[109]^. Given that extrusion-based bioprinting is the most common strategy employed in those studies we include, it is necessary to select the ideal crosslinking strategy for enhancing the mechanical and biological properties of the fabricated scaffolds.

**Table 5:** Crosslinking methods after 3D bioprinting in included studies

**Bioprinted sEVs**	**Crosslinking methods**	**Crosslinking conditions**	**Ref.**
BMSCs-sEVs	Photocrosslinking	0.25% (w/v) LAP, visible light (405 nm) for 30 s	[34]
M0-eBMP2-sEVs	N/A	N/A	[35]
BC-M0-sEVs	Ionic interaction	2% CaCl_2_ solution	[12]
hADSCs-sEVs	Chemical crosslinker	50 mM EDC, 50 mM NHS, 50 mM MES, 60% ethyl alcohol (as zero-length crosslinker), for 6 h	[36]
HUVECs-sEVs	Photocrosslinking	1 mg/mL Irgacure 2959, UV light (365 nm, 4-5 mW/cm^2^) for 5 min	[37]
M2-sEVs	Ionic interaction	100 mM CaCl_2_ solution	[38]
hUMSCs-sEVs	Photocrosslinking	0.2% (w/v) LAP, visible light (405 nm, 3 W) for 2 min	[39]
hADSCs-sEVs	Photocrosslinking	8 mg/mL Irgacure 2959, UV light (CL-1000 Ultraviolet crosslinking oven) for 20 min	[40]
hGFs-sEVs/hPDLCs-sEVs	Photocrosslinking	0.15% (w/v) LAP, LED panel (405 nm, 20 W) at 1 cm curing offset for 1 min	[16]

sEVs: Small extracellular vesicles; BMSCs: bone marrow-derived mesenchymal stem cells; M0: unpolarized macrophage; M2: anti-inflammatory macrophage; eBMP2: engineered bone morphogenetic protein 2; BC-M0-sEVs: small extracellular vesicles from M0-state RAW 264.7 cells induced by β-tricalcium phosphate bioceramic (β-TCP); hADSCs: human adipose-derived stem cells; HUVECs: human umbilical vein endothelial cells; hUMSCs: human umbilical mesenchymal stem cells; hGFs: human gingival fibroblasts; hPDLCs: human periodontal ligament cells; LAP: lithium phenyl-2,4,6-trimethylbenzoylphosphinate; EDC: 1-(3-dimethylaminopropyl)-3-ethylcarbodiimide hydrochloride; NHS: N-hydroxy-succinamide; MES: morpholine ethane sulfonic acid; LED: light-emitting diode; N/A: not applicable.

In general, crosslinking techniques can be divided into chemical (e.g., photocrosslinking, thermal crosslinking, chemical reactions, *etc*.), physical (e.g., ionic interactions and hydrogen bonds), or enzymatic types^[111]^. Among the studies this review includes, six studies employed chemical crosslinking methods (five with photocrosslinking^[16,34,37,39,40]^ and one with chemical crosslinkers^[36]^) which is more likely to fabricate a hydrogel with firm structure and stable properties^[112]^. Compared with physical methods, photocrosslinking is a mostly used and cost-effective method that can be performed under mild reaction conditions (e.g., room temperature), offering rapid curing, lower cytotoxicity, and precise spatiotemporal control^[113,114]^. Among the photoinitiators, lithium-acyl phosphinate (LAP) is mostly used due to its capability to trigger crosslinking reaction under visible light exposure (395-405 nm)^[115]^, while Irgacure 2959 is UV-sensitive^[109,114]^, thus proving a safer environment for the operator. In addition, Xu *et al*. investigated the effects of the two photoinitiators on the microstructure in 3D bioprinting of GelMA-based cellular constructs, showing that LAP-crosslinked constructs presented lower swelling ratio, stronger mechanical strength, slower degradation rate and smaller pore size^[116]^. Kang *et al*. used 50 mM 1-(3-dimethylaminopropyl)-3-ethylcarbodiimide hydrochloride (EDC), 50 mM N-hydroxy-succinamide (NHS), 50 mM morpholine ethane sulfonic acid (MES) and 60% ethyl alcohol as a zero-length crosslinker, among which EDC served as the core component for crosslinking^[36]^. It is notable that the concentration of EDC highly associates with the degree of crosslinking and the mechanical properties of the substrates^[117]^.

Physical crosslinking relies on non-covalent interactions, such as ionic interactions and electrostatic interactions^[118]^. Two studies^[12,38]^ employed the ionic interaction method for the crosslinking process, while the CaCl_2_ solution served as a crosslinker. This method has a rapid crosslinking process but also faces challenges such as poor mechanical properties and stability, possibility of metal ions release, poor reproducibility of properties and low internal crosslinking density^[115]^, which may limit its application in bone and other hard tissues. No included study employed an enzymatic reaction for crosslinking. Although the methods of crosslinking are complex, the selection should be guided by the experimental conditions and bioinks components.

Moreover, in these studies, the bioinks’ crosslinking occurs after the bioprinting process, whereas *in situ* crosslinking, which offers higher crosslinking uniformity, has been scarcely studied. Crosslinking after bioprinting places higher demands on the printability and rheological properties of bioinks, requiring sufficient support for each layer and preventing the collapse of the formed structure. Overall, the selection of crosslinking techniques must consider structural stability and bioink formulation for better functionality and clinical applicability of 3D bioprinted constructs.

## CURRENT STATE AND FUTURE DIRECTIONS OF 3D BIOPRINTED sEVs CONSTRUCTS IN PERIODONTAL REGENERATION

### Current applications of 3D bioprinted sEVs constructs in periodontal regeneration

**Figure 4:**
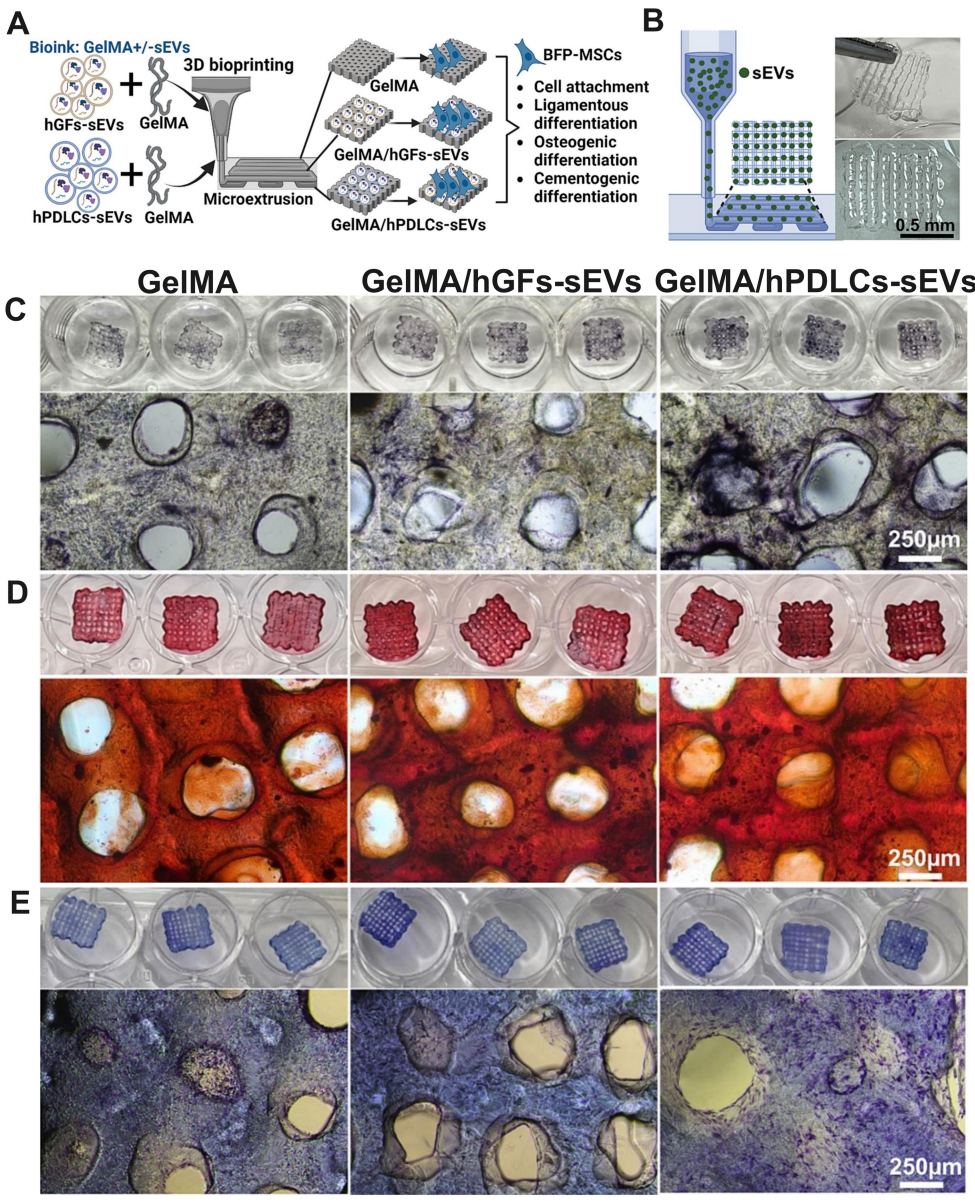
Bioprinted periodontal cell sEVs in periodontal regeneration *in vitro*. (A) Workflow for fabricating bioprinted GelMA + hPDLCs-sEVs/hGFs-sEVs constructs; (B) Demonstration of 3D bioprinted sEVs scaffolds; (C) hBFP-MSCs cultured on bioprinted sEVs constructs with osteogenic differentiation media showed enhanced alkaline phosphatase staining at 2 weeks; (D) Alizarin Red staining confirmed osteogenic differentiation; (E) Toluidine Blue staining indicated ligamentous differentiation after 2 weeks. Adopted from Ref.^[16]^. hBFP-MSCs: Human buccal fat pad-derived mesenchymal stromal cells; GelMA + hPDLCs-sEVs/hGFs-sEVs: hPDLCs-sEVs/hGFs-sEVs laden in GelMA; GelMA: gelatin methacryloyl; hPDLCs-sEVs: small extracellular vesicles derived from human periodontal ligament cells; hGFs-sEVs: small extracellular vesicles derived from human gingival fibroblasts; sEVs: small extracellular vesicles; ALP: alkaline phosphatase.

While the preceding sections could support the hypothesis that 3D bioprinted sEVs constructs could be a novel cell-free regenerative therapy with significant osteogenic, angiogenic, and immunomodulatory potential, their specific application in periodontal regeneration remains in its infancy. To date, only one pioneering investigation has directly examined 3D bioprinted constructs incorporating sEVs derived from primary periodontal cells for periodontal tissue regeneration. Han *et al*. conducted the first systematic study investigating bioprinted constructs embedded with sEVs derived from hPDLCs and human gingival fibroblasts (hGFs)^[16]^. This groundbreaking work demonstrated the feasibility of achieving controlled sEVs release from bioprinted scaffolds without requiring direct cell transplantation [Figure 4A and B]. The bioprinted constructs successfully promoted cellular adhesion and facilitated the critical processes of ligamentous, osteogenic, and cementogenic differentiation in human buccal fat pad-derived MSCs (hBFP-MSCs) *in vitro* [Figure 4C-E]. Additionally, the authors reported that the tensile modulus of the scaffolds was comparable between groups (GelMA: 15.7 ± 0.68 kPa, GelMA/hGFs-sEVs: 16.6 ± 4.5 kPa and GelMA/hPDLCs-sEVs: 15.3 ± 0.79 kPa), suggesting incorporation of sEVs might not change the mechanical property of scaffolds^[16]^. However, further validation studies are warranted, including additional testing of mechanical properties, degradation rate, swelling behaviour, and compressive strength, which would be helpful for optimizing ideal periodontal-specific bioinks in the future.

However, this single study represents only the initial exploration of this promising field. Critical knowledge gaps remain, particularly regarding *in vivo* efficacy, optimal scaffold design for periodontal-specific applications, and long-term safety profiles. The absence of comprehensive *in vivo* validation studies presents both a significant limitation and substantial opportunity for future research and clinical development in periodontal regenerative medicine.

### Future perspectives and strategic roadmap

**Figure 5:**
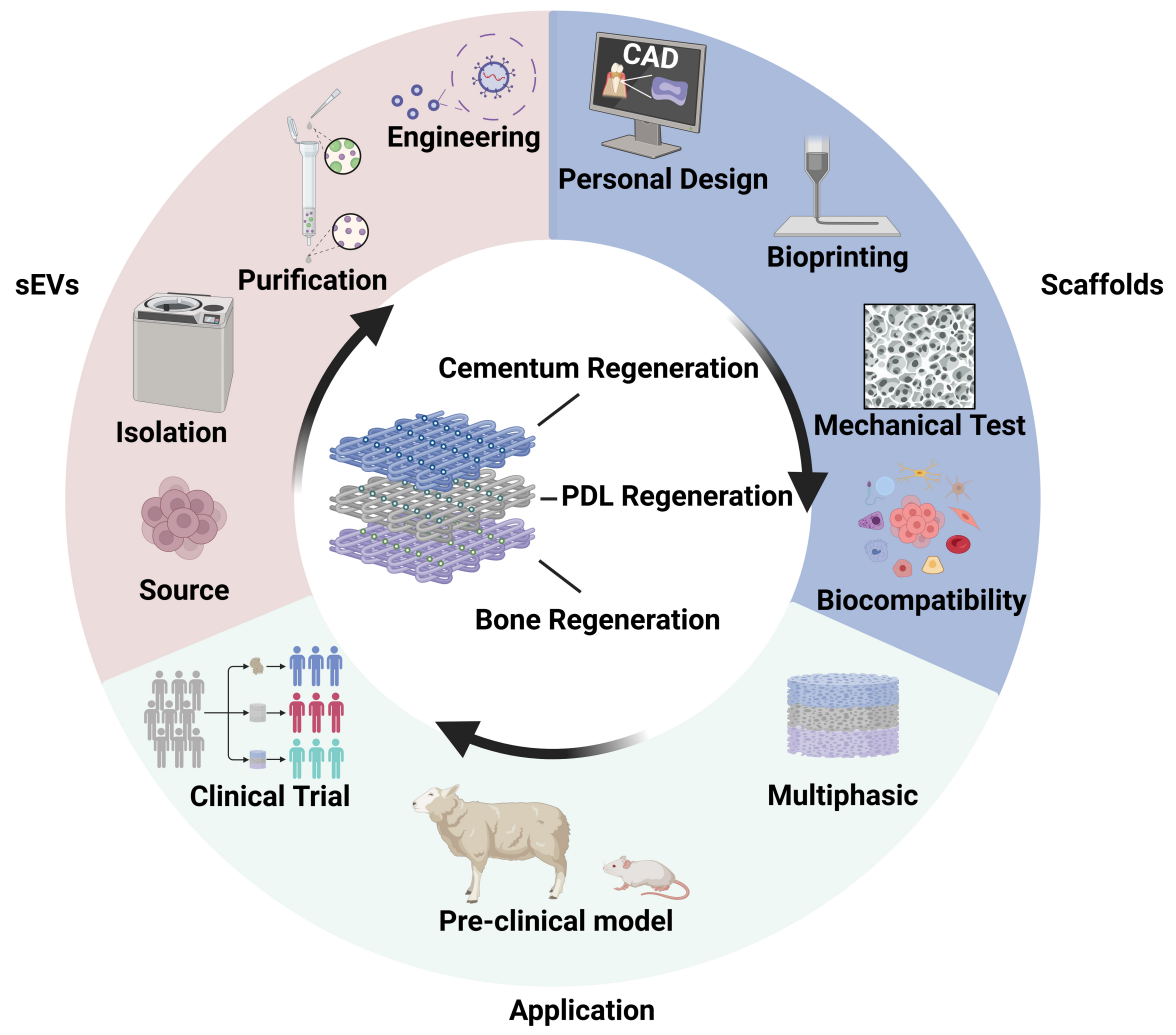
Current challenges and future directions of bioprinted sEVs. Multiphasic bioprinted scaffolds loaded with sEVs derived from various parent cells are designed to simultaneously target regeneration of cementum, PDL, and bone. Depending on the target tissue, natural sEVs are isolated and purified, and engineered sEVs are modified as needed for incorporation into bioinks. Personalized scaffold design is achieved using CAD-CAM technology. Following 3D bioprinting, constructs undergo mechanical testing and *in vitro* evaluation. Preclinical studies and clinical trials are essential prior to clinical application. Created in BioRender. Zhang, C. (2025) https://BioRender.com/iz32ou4. sEVs: Small extracellular vesicles; PDL: periodontal ligament; CAD: computer-aided design; CAM: computer-aided manufacturing; 3D: three-dimensional.

The integration of 3D bioprinting technology with sEV-based therapeutics represents a paradigm shift toward precision regenerative medicine for periodontal diseases. Yet clinical translation faces major barriers spanning the development pipeline, from sEVs production to clinical evaluation. Figure 5 illustrates a future workflow of multiphasic 3D-bioprinted scaffolds targeting functional periodontal regeneration, as well as current major challenges.

The clinical evidence base is extremely limited, with only a single investigation to date examining bioprinted hPDLC-derived sEVs *in vitro*^[16]^, and no animal studies have validated efficacy in clinically relevant periodontal defect models. Protocols for sEVs isolation, characterization, and quality control are unstandardized, and optimal bioprinting parameters for periodontal use are undefined. Manufacturing hurdles include the need for GMP-compliant workflows and clear regulatory guidance, while limited clinical trial data and uncertain economic feasibility hinder adoption. Overcoming these challenges through coordinated scientific, regulatory, and commercial efforts will be essential to unlock the clinical potential of 3D bioprinted sEVs therapeutics.

#### sEVs heterogeneity and current limitations in production

The inherent heterogeneity of sEVs populations - varying in size distribution, molecular cargo, and biological functions - necessitates the development of standardized protocols for isolation, characterization, purification, and storage^[119]^. Among the nine studies reviewed, isolation methodologies varied significantly, with two studies employing SEC^[16,35]^, while the remaining studies used UC. Current approaches face critical limitations, including low throughput processing, where UC-based isolation proves time-intensive and may result in contamination with non-EV components. Additionally, inconsistent isolation protocols contribute to batch-to-batch variability in sEVs quality and therapeutic efficacy, while limited standardization of purity assessment and functional characterization creates significant quality control gaps.

To address these challenges, multiple isolation methods should be employed in combination to achieve higher purity and improved functionality^[90]^. Accurate quantification protocols, including comprehensive assessment of total protein content, purity indices, and particle number, should become mandatory for clinical applications^[120]^. Furthermore, the development of scalable, GMP-compliant production workflows is essential for generating clinical-grade sEVs that meet regulatory standards. These standardization efforts will be crucial for establishing the reproducibility and reliability necessary for successful clinical translation.

Beyond production standardization, several sEVs modification strategies have emerged to enhance therapeutic potential by improving circulation time, targeting specificity, and biomaterial integration. Co-incubation methods offer a simple, cost-effective approach for loading therapeutic molecules, while chemical transfection provides enhanced loading efficiency for specific cargo molecules. Electroporation enables precise control over sEVs cargo modification, and sonication techniques improve sEVs permeabilization for cargo loading^[121-123]^. These modification strategies are particularly valuable for developing advanced sEV-based therapies specifically designed for the complex regenerative requirements of periodontal tissues, where multiple tissue types must be regenerated in a coordinated manner.

#### Scaffold design challenges and safety requirements

Accurately replicating the compartmentalized and hierarchical architecture of periodontal tissues represents one of the most significant technical challenges in this field^[124-126]^. Bioprinted scaffolds must simultaneously address multiple design criteria to ensure both safety and functional efficacy. From a safety perspective, scaffolds require biocompatible materials that are non-toxic and non-immunogenic, predictable degradation rates that avoid cytotoxic product release^[127]^, and gradual pore formation through degradation-mediated processes to facilitate oxygen and nutrient diffusion while enabling sustained sEVs release^[78]^. The functional architecture requirements are equally demanding, requiring optimization of nano- and micropores (ranging from < 200 nm to 100-1,000 nm) for optimal sEVs depot function^[128]^. The scaffolds must also support diverse cellular activities through tissue-specific porosity, including larger pores (> 5 μm) for neovascularization, intermediate pores (5-15 μm) for fibroblast ingrowth, and substantial pores (100-350 μm) for bone regeneration^[129]^. Achieving multi-tissue integration requires hierarchical organization with spatially organized scaffold regions for different periodontal tissues, mechanical property gradients that match the diverse requirements of soft and hard periodontal tissues, and interface optimization to ensure seamless integration between different tissue compartments.

#### Functional architecture and multi-tissue integration needs

The field currently faces several critical limitations that must be addressed for successful clinical translation. The limited clinical evidence base, consisting of only one investigation examining bioprinted hPDLCs-sEVs^[16]^, represents a significant knowledge gap. The absence of comprehensive animal studies demonstrating efficacy in periodontal defect models further constrains our understanding of *in vivo* performance^[31,130]^. Additionally, the essential requirement for using primary cells rather than immortalized cell lines for clinically relevant sEVs production adds complexity to manufacturing processes. The clinical trial landscape remains sparse, with only one registered randomized controlled trial identified on the WHO platform and no published clinical outcomes data available. This limited clinical experience is compounded by regulatory gaps, as current guidance for sEV-based bioprinted products remains underdeveloped.

#### Strategic roadmap: a phased approach

Several technical issues remain to be addressed, including large-scale sEVs production, bioprinting precision and long-term *in vivo* safety. As for sEVs isolation, TFF has been employed for large-scale sEVs enrichment for its suitability that meets the requirements of GMP grade^[91,92]^, and will have more advantages when combined with SEC for producing clinical-grade sEVs with higher purity also without significant loss in particle yield or changes in size^[131,132]^. It has also been reported that biomaterials could influence the yield and composition of cell-derived sEVs, with enhanced yield potentially resulting from the 3D growth mode of cells seeded on the scaffolds^[58]^. In addition, bioinks with tunable rheological properties and batch-to-batch replicability are critical in improving the precision of bioprinting. At the same time, more efforts should be made in introducing emerging strategies (e.g., predictive models), unified metrics and standardized protocols^[133,134]^. Finally, systematic preclinical studies are needed to assess potential safety concerns and the long-term stability of bioprinted sEVs constructs.

Future research priorities must be strategically organized across multiple timeframes to ensure systematic progress toward clinical translation. In the immediate term (1-3 years), the field requires comprehensive *in vivo* studies using well-designed animal models with appropriate periodontal defect characteristics, systematic optimization studies evaluating bioprinting parameters and sEVs formulations, thorough safety assessments including biocompatibility and toxicology evaluations, and development of consistent sEVs production and characterization protocols. Medium-term objectives (3-7 years) should focus on preclinical validation through large animal studies demonstrating both efficacy and safety, collaborative development of regulatory pathways with appropriate agencies, establishment of commercial-scale manufacturing capabilities, and advancement of personalized approaches including patient-specific scaffold design and sEVs customization.

Long-term goals (7-15 years) encompass the conduct of rigorously designed Phase I, II, and III clinical studies, development of commercial clinical products and standardized treatment protocols, integration of these technologies into routine periodontal care practices, and ultimate achievement of fully personalized treatment approaches based on patient-specific parameters. This staged approach ensures that fundamental scientific and technical challenges are addressed before advancing to more complex clinical applications, while maintaining focus on the ultimate goal of transforming periodontal regenerative medicine through precision, cell-free therapeutic strategies.

## CONCLUDING PERSPECTIVES AND FUTURE POTENTIAL

The synergetic potential of 3D bioprinting technology with sEV-based therapeutics may represent a transformative approach to periodontal regenerative medicine, with the potential to deliver precisely targeted, patient-specific treatments while overcoming many of the limitations of cell-based therapies. Although clinical applications remain in their infancy, early proof-of-concept studies have laid a solid foundation for this cell-free regenerative strategy. It is promising in matching the regenerative requirements of cementum, PDL, and alveolar bone, while also enabling scalable, standardized manufacturing, and supporting personalization through specific scaffold architecture and sEVs cargo.

Realizing this potential will require overcoming key translational hurdles. Integration of advanced bioprinting workflows with optimized, GMP-compliant sEVs production is essential, as is early engagement with regulatory agencies to define approval pathways. Robust preclinical models and well-designed clinical trials must establish both safety and efficacy, while cost-effective scale-up will determine commercial viability.

If these challenges are met, 3D bioprinted sEVs therapeutics could shift periodontal care from reactive disease management to proactive, precision regeneration, enabling durable tissue restoration and improved long-term outcomes. Beyond dentistry, the principles and platforms may also accelerate the broader adoption of sEV-integrated bioprinting across regenerative medicine, representing a step toward customizable, cell-free therapeutics.

## DECLARATIONS

### AI and AI-assisted tools statement

During the preparation of this work, the authors used Artificial intelligence tools (ChatGPT) for basic grammar and spelling checking. The authors reviewed and edited all AI-generated content and accept full responsibility for the published material.

### Copyright

© The Author(s) 2025.
